# Arrhythmias in Rheumatoid Arthritis: A Call for a Multidisciplinary Team

**DOI:** 10.3390/life15091426

**Published:** 2025-09-11

**Authors:** Veronica Ungurean, Diana Elena Costan, Monica Claudia Dobos, Anca Ouatu, Paula Cristina Morariu, Alexandru Florinel Oancea, Maria Mihaela Godun, Diana-Elena Floria, Dragos Traian Marcu, Genoveva Livia Baroi, Silviu Marcel Stanciu, Anton Knieling, Daniela Maria Tanase, Codrina Ancuta, Mariana Floria

**Affiliations:** 1 Department of Medical Sciences II, “Grigore T. Popa” University of Medicine and Pharmacy, 700115 Iasi, Romania; ungurean.veronica@yahoo.com (V.U.); costan.diana0198@yahoo.com (D.E.C.); monicabalac329@yahoo.com (M.C.D.); 2 Department of Rheumatology, Clinic Rehabilitation Hospital, 700661 Iasi, Romania; 3 Department of Medical Sciences I, “Grigore T. Popa” University of Medicine and Pharmacy, 700115 Iasi, Romania; morariu.paula-cristina@email.umfiasi.ro (P.C.M.); alexandru.oancea@umfiasi.ro (A.F.O.); godun.maria-mihaela@d.umfiasi.ro (M.M.G.); diana-elena.iov@d.umfiasi.ro (D.-E.F.); marius-traian-dragos.dm-marcu@umfiasi.ro (D.T.M.); daniela.tanase@umfiasi.ro (D.M.T.); floria.mariana@umfiasi.ro (M.F.); 4 Department of Internal Medicine, Sf. Spiridon County Clinical Emergency Hospital, 700111 Iasi, Romania; 5 Department of Surgery, Faculty of Medicine, “Grigore T. Popa” University of Medicine and Pharmacy, 700115 Iasi, Romania; livia.baroi@umfiasi.ro; 6 Department of Internal Medicine and Gastroenterology, Carol Davila University of Medicine and Pharmacy, Central Military Emergency University Hospital, 010825 Bucharest, Romania; silviu.stanciu@umfcd.ro; 7 Discipline of Forensic Medicine, Faculty of Medicine, “Grigore T. Popa” University of Medicine and Pharmacy Iasi, 16 Universitatii Street, 700115 Iasi, Romania; anton.knieling@umfiasi.ro

**Keywords:** rheumatoid arthritis, arrhythmias, atrial fibrillation, ventricular ectopic beats, epidemiology, csDMARDs, bDMARDs, tsDMARDs

## Abstract

Background: Rheumatoid arthritis is the most prevalent systemic inflammatory disease, mainly affecting the synovial tissue of small and large joints, also associated with significant extra-articular manifestations. Throughout the progression of the disease, cardiac structures may be affected, including the conducting system, myocardium, endocardium, coronary arteries, and valves, potentially resulting in a higher incidence of cardiac arrhythmias. Methods: We performed a narrative review of the most recent studies that highlight the epidemiology, pathophysiology, diagnosis, and management of arrhythmias occurring in patients with rheumatoid arthritis. Furthermore, we examined the impact of disease-modifying antirheumatic drugs (DMARDs)—including conventional synthetic (csDMARDs), biologic (bDMARDs), and targeted synthetic agents (tsDMARDs)—on cardiac electrophysiology. Results: Cardiac immune cells may influence arrhythmogenesis through non-canonical and inflammatory mechanisms by modifying myocardial tissue architecture or by interacting with cardiomyocytes, potentially altering their electrical function. Conclusions: This review emphasizes the essential role of a multidisciplinary approach integrating rheumatology and cardiology expertise in the screening and management of arrhythmias in patients with rheumatoid arthritis.

## 1. Introduction

Rheumatoid arthritis (RA) is the most prevalent systemic inflammatory rheumatic disease, targeting mainly the synovial tissue of small and large joints, mediated by the interaction between T lymphocytes, B lymphocytes, and synovial fibroblasts. Throughout the progression of the disease, cardiovascular involvement is of particular concern, as RA can affect all cardiac structures—including the conducting system, myocardium, endocardium, coronary arteries, and valves—thereby contributing to an increased risk of cardiac arrhythmias [1].

Preclinical and clinical data underscore the critical role of the immune system in the pathophysiology of arrhythmias. Cardiac immune cells were found to contribute to arrhythmogenesis through both inflammatory mechanisms and non-canonical functions. These processes can disrupt myocardial tissue architecture or directly interact with cardiomyocytes, thereby altering their electrical properties and contributing to abnormal cardiac conduction [2].

In current clinical practice, RA is recognized as a complex systemic disease with multisystem involvement. A multidisciplinary approach is therefore essential, as RA, while primarily affecting the synovial tissue of both small and large joints, can also lead to significant extra-articular complications, including cardiac manifestations such as arrhythmias.

This review aims to integrate insights from recent literature in order to provide a comprehensive overview of arrhythmia management in RA patients. In addition to highlighting the essential role of interdisciplinary collaboration in improving patient outcomes, this paper covers topics ranging from epidemiology and pathophysiological mechanisms to diagnostic strategies and cardiovascular impact of antirheumatic drugs.

## 2. Materials and Methods

This paper was designed as a narrative review, aiming to synthesize the latest literature on the epidemiology, pathophysiology, diagnosis and management of arrhythmias found in patients with RA, as well as the impact of disease-modifying antirheumatic drugs (DMARDs)—including conventional synthetic (csDMARDs), biologic (bDMARDs), and targeted synthetic agents (tsDMARDs)—on cardiac electrophysiology.

The literature search was conducted using a structured query of PubMed and Google Scholar, combining the following descriptors: “rheumatoid arthritis”, “arrhythmias”, “atrial fibrillation”, “ventricular ectopic beats”, “epidemiology, pathophysiology”, “autonomic nerve system”, “renin-angiotensin system”, “endothelial dysfunction”, “epicardial tissue adiposity”, “inflammation”, “csDMARDs, bDMARDs and tsDMARDs”. Articles published in the last ten years (2015–2025) were prioritized. A selection of older papers was included when these provided foundational knowledge or established clinical guidelines critical to understanding the long-term development of cardiovascular complications.

**Figure 1:**
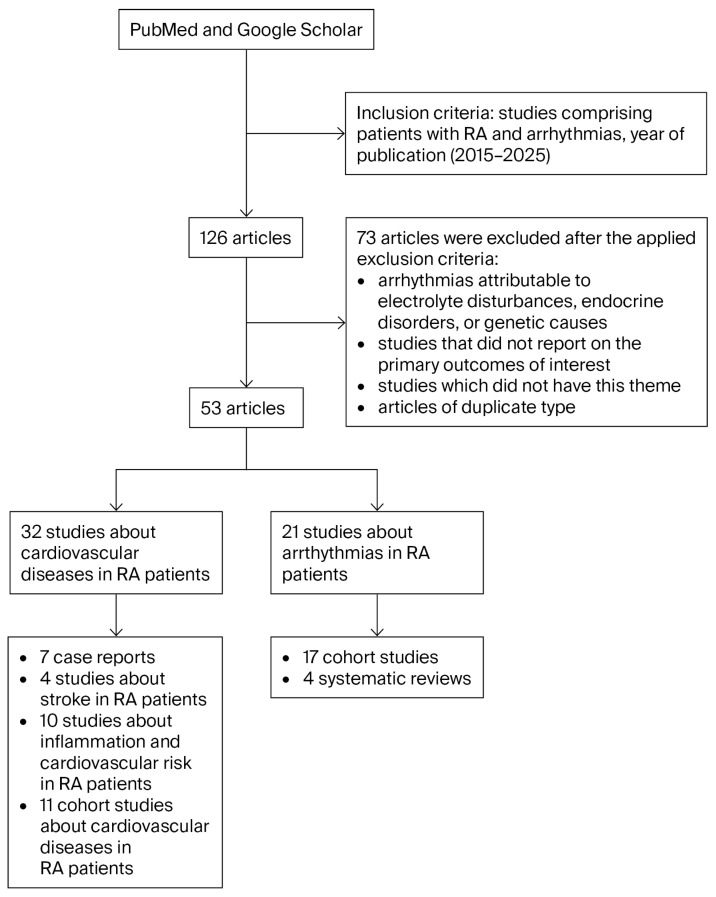
Flow chart of narrative review process (RA, rheumatoid arthritis).

Inclusion criteria were defined based on the year of publication, studies comprising patients with RA and cardiac arrhythmias. Although the review focused on English-language literature, translated versions of relevant articles were also considered. Exclusion criteria were applied in a limited manner: studies involving patients with comorbid conditions beyond the scope of this review, arrhythmias attributable to electrolyte disturbances, endocrine disorders, or genetic causes, studies that did not report on the primary outcomes of interest, studies that did not have this theme, or duplicate articles were excluded (Figure 1). Given the specific focus on arrhythmias in the context of rheumatoid arthritis, no restrictions were imposed on the number of studies included.

## 3. Epidemiology of Arrhythmias in Rheumatoid Arthritis

### 3.1. General Prevalence of Arrhythmias in Rheumatoid Arthritis

Autoimmune diseases are linked with increased cardiovascular morbidity and mortality, with cardiovascular disease accounting for more than 50% of premature deaths in affected individuals. In RA patients, atrial fibrillation (AF) was found to be 40% more common compared to the general population. According to a Danish cohort study, 18,247 of the 4,182,335 participants (mean onset age 59.2 years, median follow-up 4.8 years) developed RA. Atrial fibrillation was diagnosed in 156,484 individuals, including 774 with RA, corresponding to event rates of 8.2 vs. 6.0 per 1000 person-years and an adjusted incidence rate ratio of 1.41 (95% CI, 1.31–1.51). In RA patients, AF was found to be 40% more common compared to the general population (age and sex matched event rates of 8.2 and 6.0 per 1000 person years). Across all age and sex groups, there were higher relative risks, which were more noticeable in younger patients, but older people had larger absolute risk differences. Women had slightly higher relative risks than men, with AF risk most pronounced in younger patients. The absolute risk attributable to RA ranged from 70% in the youngest to 25% in the oldest age group. Atrial fibrillation can occur at any stage of the disease progression, sometimes even presenting as the first clinical manifestation [3,4].

The pathophysiology of AF is closely linked to systemic inflammation, which leads to an increase in circulating inflammatory markers. This effect appears particularly pronounced in younger females (<50 years) with sedimentation rates exceeding 60 mm/h or elevated levels of anti-TNF-α antibodies [5]. However, despite this observation, a Mendelian randomization study found no causal relationship between RA and AF, suggesting that this association may be due to shared risk factors [6].

Other arrhythmias, such as ventricular tachycardia (VT), were reported, but usually associated with treatments like methotrexate (MTX) and infliximab [7]. A comprehensive analysis of the National Inpatient Sample Database (2015–2019) involving 9370 RA patients found that 25.7% had concomitant arrhythmias. More specifically, 12% presented with supraventricular tachycardia (SVT), 73.4% with AF, 9.8% with atrial flutter (AFl), and 6.4% with VT. Based on these findings, patients with RA have a 25.7% risk of developing arrhythmias [8].

Moreover, patients with RA have a two-fold higher risk of sudden cardiac death compared to healthy controls. This is most often due to VT or ventricular fibrillation and occurs in about 0.2% of the general population annually [9].

### 3.2. Autoantibody-Associated Arrhythmias in Rheumatoid Arthritis

The main features that distinguish RA from other inflammatory joint diseases are rheumatoid factor (RF) and anti-citrullinated protein antibodies (ACPA). These autoantibodies are found in the blood and joint fluid of 60–70% of patients in early stages, often years before symptom onset. Seropositive RA is characterized by the presence of RF and/or ACPA and is now widely recognized as a distinct subtype, separate from seronegative RA [10]. Nevertheless, up to 20% of RA cases remain seronegative [11].

A 2021 study including women with moderate-to-high activity seropositive RA identified arrhythmias in 56.25% of patients, most commonly sinus rhythm abnormalities (31.25%), including tachycardia (18.75%), bradycardia (6.25%), and sinus arrhythmia (6.25%). Premature supraventricular and ventricular beats were present in 12.5%, and left anterior bundle branch block was found in 12.5%. Myocardial repolarisation disturbances suggestive of silent ischemia were observed in 18.75% of participants, exclusively in those with RA duration over 10 years. These findings support the use of Holter ECG monitoring in seropositive RA to detect asymptomatic cardiovascular complications [12].

Rheumatoid factor is an autoantibody directed against the Fc region of immunoglobulin, with a sensitivity of 60–80% and a specificity of 85% for RA, routinely used for diagnostic purposes in current clinical practice [13,14]. A study by Kumar et al. found that the presence of RF has a significant impact on cardiovascular involvement in patients with RA. Electrocardiogram (ECG) abnormalities were the most common cardiovascular manifestations, present in 58.3% of RA patients (82.9% RF-positive) [13].

Anti-citrullinated protein antibodies, which target citrullinated peptides, are considered more specific serological markers compared to RF. Anti-citrullinated protein antibody testing has been incorporated into the 2010 revised RA classification criteria established by the American College of Rheumatology (ACR) and the European League Against Rheumatism (EULAR) [14]. Anti-citrullinated protein antibodies may contribute to subclinical cardiac changes in patients with RA; more specifically, elevated ACPA levels were found to enhance the correlation between high-sensitivity troponin T and left ventricular mass index, indicating a potential role of these autoantibodies in myocardial remodelling.

No direct association has been described between ACPA and the prevalence of arrhythmias. However, current evidence supports the hypothesis that ACPA may contribute to subclinical cardiac injury and structural alterations [13]. Some RA patients develop eccentric cardiac remodelling with reduced relative septal thickness, a change that correlates with elevated levels of ACPA and γ-globulin, suggesting a potential correlation with disease severity [7].

Potential known triggers for ACPA production are filaggrin, type II collagen, α-enolase, fibrinogen, and vimentin [15]. In a cross-sectional study of 135 seropositive RA patients with no history of cardiovascular disease, plasma levels of anti-modified citrullinated vimentin (anti-MCV) antibodies were found to correlate inversely with left ventricular ejection fraction, with a stronger negative correlation (*p* < 0.001) than that observed for general ACPA (*p* = 0.019). These findings suggest that anti-MCV could serve as a promising biomarker for early screening of cardiac systolic dysfunction in RA [16].

Anti-Ro antibodies, present in 5–15% of RA patients, are linked to cardiac arrhythmias, such as QTc prolongation. These antibodies downregulate L-type calcium channels, promoting conduction defects, but also inhibiting the hERG/IKr current by binding to Kv11.1 channels, leading to prolonged repolarization. Anti-Ro antibodies were positive in 60% of patients with torsades de pointes or long QT syndrome (LQTS), often in the absence of an associated connective tissue disease, suggesting a complex proarrhythmic role through multiple ion channel interactions [9]. Additionally, data suggest that anti-Ro antibodies (particularly the 52 kDa subtype) can contribute to LQTS and ventricular arrhythmias [17].

**Figure 2:**
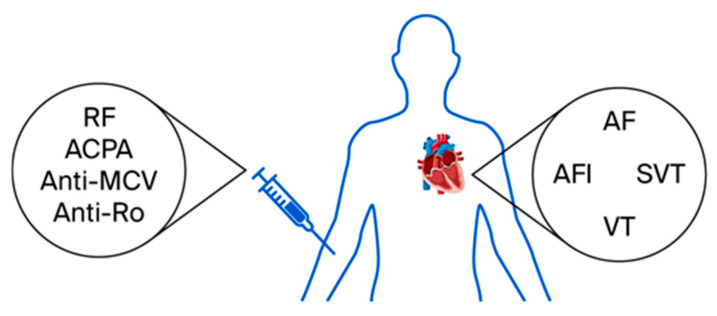
Illustration of arrhythmias in RA patients based on antibody profiles (ACPA, Anti-Citrullinated Protein Antibodies; AF, Atrial Fibrillation; AFl, Atrial Flutter; anti-MCV, Anti-Modified Citrullinated Vimentin Antibodies; RF, Rheumatoid Factor; VT, Ventricular Tachycardia; SVT, Supraventricular Tachycardia).

The relationship between RA-associated antibodies and the prevalence of arrhythmias remains insufficiently characterized (Figure 2). Emerging evidence highlights intriguing associations that warrant further investigation.

## 4. Pathophysiology of Arrhythmias in Rheumatoid Arthritis

### 4.1. Atrial and Ventricular Remodelling

The immunological pathogenesis of RA-associated AF appears to be driven by atrial fibrosis and structural remodelling, primarily mediated by the hyperactivation of resident cardiac macrophages and polymorphonuclear neutrophils (PMNs) [18]. More specifically, the structural and functional changes may be driven by chronic inflammation, particularly IL-6, and cardiomyocyte apoptosis, triggered by inflammation and oxidative stress, contributing to a pro-arrhythmic substrate. Extensive fibrosis disrupts normal atrial architecture and electrical conduction, increasing susceptibility to AF. Experimental models of collagen-induced arthritis show heightened AF inducibility and duration, supporting an inflammatory link [9].

Wang et al. examined the role of various inflammatory and immunological markers found in RA patients with and without AF. Significantly increased levels of Th1, Th17, and Th1/Treg cells were observed in patients with RA-associated AF. Th1 cells produce interferon-gamma (IFN-γ) and interleukin-2 (IL-2), which may contribute to atrial remodelling by stimulating proinflammatory cytokine secretion from macrophages. Additionally, elevated levels of interleukin-17A, secreted by Th17 cells, have been associated with the pathogenesis of AF through neutrophil-mediated inflammation and myocardial fibrosis [18]. The activation of complement locally leads to tissue damage, necrosis of myocytes and subsequent fibrosis, which triggers remodelling in the atrium. These structural changes result in alterations in conduction patterns and varying refractory periods, often presenting as prolonged P wave duration and increased P wave dispersion (Pd) [1].

Chronic inflammation in RA contributes to ventricular morphological changes, such as the development of fibrosis, which may promote arrhythmias. Overt systolic dysfunction is uncommon, but subtle diastolic impairment is frequent and correlates with disease activity. Left ventricular (LV) remodelling is common in RA patients without clinically apparent heart failure (HF). Proinflammatory cytokines such as TNFα and IL-1β have been shown to downregulate and disrupt connexin distribution in cardiomyocytes. Although ventricular connexin expression has not been directly studied in RA patients, evidence from Lazzerini et al. demonstrates an inverse association between atrial connexin levels and serum IL-6 in patients with inflammatory diseases. These findings suggest that chronic inflammation in RA may contribute to ventricular arrhythmogenesis via connexin downregulation [9]. In a study of 158 RA patients without clinical HF, transthoracic echocardiography over 4–6 years showed an increase in LV remodelling, from 40% to 60%. Elevated IL-6 levels were associated with concentric remodelling, and tocilizumab use correlated with increased relative wall thickness at baseline. These findings highlight IL-6 as a potential biomarker of LV remodelling in RA, warranting further prospective studies on its progression and modulation through IL-6 inhibition [19].

### 4.2. Autonomic Nervous System

Autonomic nervous system dysfunction in RA (evident in about 60% of patients) is linked to the neurotoxic impact of chronic systemic inflammation, as well as side effects of certain therapeutic agents. The primary pattern involves disrupted cardiovascular reflexes and abnormal heart rate variability, reflecting decreased parasympathetic activity and increased sympathetic tone. This imbalance can lead to ectopic atrial beats, poor heart rate regulation and episodes of atrial tachycardia [7]. Arrhythmias may arise from various electrophysiological disturbances, such as increased automaticity, delayed afterdepolarizations due to excess intracellular calcium, early afterdepolarizations caused by prolonged action potential duration, and the formation of re-entrant circuits [9].

The regulation of cardiovascular, fluid, and energy balance relies on the coordinated interaction between the neuroendocrine and autonomic nervous systems, controlled by central neural networks. In the progression of HF, these regulatory networks become impaired, leading to autonomic dysfunction. Chronic overactivation of G protein-coupled receptor kinase 2 (GRK2) has been linked to cardiac dysfunction and remodelling, primarily through β1-adrenergic receptor (β1AR) desensitization. Prolonged exposure to high epinephrine levels induces GRK2-mediated β1AR phosphorylation, receptor internalization, and reduced cardiomyocyte contractility. GRK2 inhibitors are thus proposed as potential therapeutic agents capable of not only mitigating arthritis-related inflammation but also restoring β1AR sensitivity and preventing cardiac dysfunction [20].

### 4.3. Renin–Angiotensin System

The Renin–Angiotensin system has been traditionally characterized as a hormonal cascade primarily responsible for regulating blood pressure and maintaining fluid-electrolyte homeostasis, playing a central role in cardiovascular and renal physiology. In patients with RA, elevated angiotensin-converting enzyme (ACE) levels have been detected in synovial fluid, implicating the RAS pathway in joint degradation [21]. Angiotensin II (Ang II) levels are significantly higher in active disease compared to the patients in remission, promoting AF by driving inflammation, epicardial fat accumulation, and electrical remodelling of the heart. The Renin–Angiotensin system blockade therapy has been shown to reduce the relative risk of recurrent AF by 39% [22].

Some studies explored the effects of biologic and targeted synthetic DMARDs on ACE and ACE 2 levels. Janus kinase (JAK) inhibition may lead to increased serum ACE levels and a temporary rise in ACE 2 activity in patients with RA, suggesting a redistribution and shedding of renin–angiotensin–aldosterone system (RAAS) components between the synovium and the bloodstream. Baseline levels of ACE and ACE 2 appear to be associated with disease activity, inflammatory markers such as ESR, and indicators of autoimmunity like RF. Moreover, baseline autoantibody levels (RF, ACPA) may be key determinants of how tofacitinib modulates the RAAS. These changes in ACE and ACE2 dynamics may partially explain the cardiovascular benefits observed with targeted synthetic DMARDs in RA patients [23].

### 4.4. Endothelial Dysfunction

The endothelium, a specialized epithelial layer lining blood vessels, lymphatics, and the heart, functions as a key endocrine organ regulating vascular tone, coagulation, permeability, and cell adhesion. Endothelial dysfunction disrupts these functions and contributes to disease pathogenesis. During inflammation, endothelial cells exhibit two activation types: a rapid, transient type I and a delayed, sustained type II. This increases permeability, stimulates cytokine and enzyme release, and promotes leukocyte adhesion via upregulated adhesion molecules [23].

Endothelial dysfunction is prevalent in RA, even in the absence of clinically apparent cardiovascular disease, and involves both macrovascular and microvascular compartments. Macrovascular dysfunction can be evaluated through flow-mediated dilation and biomarkers such as asymmetric dimethylarginine, matrix metalloproteinases, and toll-like receptors. Microvascular dysfunction is characterized by inflammatory activation, reduced capillary density, impaired vasodilation, and defective angiogenesis [24]. In inflammatory diseases like RA, high levels of proinflammatory molecules trigger endothelial activation and dysfunction, promoting leukocyte infiltration into the heart tissue. Oxidative stress reduces nitric oxide availability, lowering cGMP and PKG levels in heart cells, which leads to cardiac hypertrophy and increased resting tension. Additionally, MHCII high macrophages, activated by IL-10, release osteopontin and TGF-β, stimulating fibroblast growth and collagen buildup. Taken together, these changes increase heart stiffness and contribute to diastolic dysfunction [25].

A 2022 study found that MTX can improve endothelial function in patients with early RA by reducing inflammation and supporting vascular repair. The mechanism involves lowering the activation of endothelial cells caused by TNF-α, specifically by regulating molecules like VCAM-1, ICAM-1, and E-selectin. MTX also prevents cell death, promotes healthy cell growth, and reduces proinflammatory cytokines such as IL-6 and MCP-1. These positive effects become noticeable after three months of treatment and continue with long-term use [26].

### 4.5. Epicardial Tissue Adiposity and Inflammation

Obesity, characterized by a chronic low-grade systemic inflammatory state, leads to adipocyte hypertrophy and a hypoxic environment that triggers genetic mutations and alters the regulation of pro- and anti-inflammatory mediators. This inflammatory state is associated with an increased risk of atrial and ventricular arrhythmias, with a 65% higher risk of AF in individuals with a BMI > 30 kg/m^2^ compared to those with a normal BMI. This increased risk is linked to electrophysiological remodelling, including atrial volume expansion, conduction abnormalities, and upregulation of pro-fibrotic mediators, creating a proarrhythmogenic substrate [24].

Epicardial adipose tissue (EAT) is a fat layer located between the myocardium and the visceral pericardium, serving as a potential source of inflammatory mediators. Previous research has found that the thickness of EAT in patients with RA is greater than in the control population. In patients with RA and AF, the LA diameter positively correlates with the EAT/BMI ratio, a relationship not observed in AF patients without RA. Previous reports have shown extensive atrial fibrosis in RA patients, which reduces LA compliance and leads to dilation. Additionally, RA patients have more pericardial adipose tissue around the LA compared to those without RA [25]. Chahine et al. demonstrated that LA epicardial adipose tissue is independently associated with LA volume and fibrosis and suggested that EAT may promote atrial structural remodelling via paracrine inflammatory factors [25]. EAT thickness correlates with P-wave duration and LA diameter in morbidly obese patients. In healthy individuals with normal atrial dimensions, the relationship between EAT and P-wave duration likely indicates slowed atrial conduction. However, in morbidly obese patients, P-wave prolongation is partly attributed to atrial enlargement. In patients with the largest EAT volumes, the PR interval was 10 to 16 milliseconds longer compared to those with the smallest EAT volumes. Observational studies have shown that a prolonged PR interval is associated with an increased incidence of AF, HF, and mortality. Additionally, high EAT volumes are also linked to a higher incidence of AF. Prolonged QRS duration, linked to a higher BMI, indicates slowed ventricular conduction and may result from hypertrophy. In a study of 3087 healthy subjects, higher EAT volume was associated with a 6.7 millisecond increase in QRS duration for those above the 95th percentile compared to those below the 5th percentile, after adjusting for covariates. Research on the relationship between EAT volume and the QT interval, as well as its dispersion (QTd), is limited. One study reported a negative association between EAT volume and QTc [26].

Higher adiponectin levels are independently associated with an increased risk of AF, particularly in older individuals. Circulating adiponectin levels are significantly elevated in RA patients, where they contribute to the proinflammatory processes involved in the pathogenesis of the disease. EAT thickness correlated positively with the severity of RA [22].

Systemic inflammatory disorders can lead to alterations in the coronary microcirculation, resulting in microvascular dysfunction and myocardial fibrosis. Since the epicardium and myocardium are interconnected through unobstructed microcirculation, the release of proinflammatory adipocytokines (TNF-α, IL-1β, IL-6) can cause microcirculatory damage and fibrosis in the adjacent myocardium, particularly in the atrial myocardium, which may contribute to the development of AF [27].

## 5. Effects of Antirheumatic Drugs on Cardiac Rhythm

Ongoing advancements in drug design have significantly enhanced pharmacological strategies for treating RA. These developments have led to therapies that effectively alleviate symptoms, slow disease progression, and reduce complications. According to the most recent ACR and EULAR guidelines, current RA management should focus on two main approaches: symptomatic treatment using nonsteroidal anti-inflammatory drugs (NSAIDs) and glucocorticoids (GCs), and disease-modifying therapy through the use of DMARDs. Initiating treatment at the earliest possible stage is essential. Due to their delayed onset of action, from 6 weeks to 6 months, DMARDs should be introduced promptly. These are classified into three categories: conventional synthetic (csDMARDs), biologic (bDMARDs), and targeted synthetic (tsDMARDs) [28].

### 5.1. csDMARDs

In recent years, the management of RA has emphasized early intervention with csDMARDs. Methotrexate is used as the unequivocal first-line agent to reduce inflammation, slow radiographic progression, and improve long-term outcomes. In cases of MTX intolerance or contraindication, leflunomide, hydroxychloroquine (HCQ), or sulfasalazine may be suitable alternatives [29].

**Figure 3:**
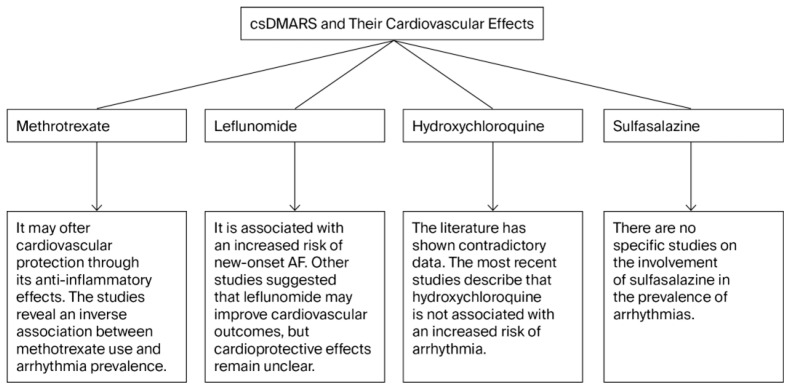
Summary of cardiovascular effects and possible correlations with development of arrhythmias for each conventional synthetic disease-modifying antirheumatic drugs (csDMARDs) (AF, atrial fibrillation).

The cardiovascular effects and possible correlations with arrhythmias for each csDMARDs are summarized in Figure 3.

#### 5.1.1. Methotrexate

Methotrexate is a folate antagonist that exerts anti-proliferative, anti-metabolic, and anti-inflammatory effects by reducing proinflammatory cytokines and limiting the infiltration of immune cells like neutrophils, monocytes, mast cells, Th cells, and B cells in the RA synovium [30].

A retrospective cross-sectional study investigated the potential association between intermittent treatment with MTX and cardiovascular risk in RA patients. The analysis compared two groups: individuals who had ceased MTX therapy prior to enrolment (MTX 0) and those actively receiving the drug at the time of data collection (MTX 1). Findings revealed a higher incidence of arrhythmias in the MTX 0 group (9.0%) compared to the MTX 1 group (4.9%), indicating that discontinuation of MTX may be associated with a significantly elevated risk of cardiovascular complications [31].

Additionally, a retrospective case–control study involving 405 patients with RA found that those who experienced cardiovascular events, including arrhythmias, were significantly less likely to have been treated with MTX (*p* = 0.003). These results suggest an inverse relationship between MTX use and the prevalence of arrhythmias. These findings support the concept that MTX may have a protective cardiovascular effect, likely due to its anti-inflammatory properties [32].

#### 5.1.2. Leflunomide

Leflunomide is a non-biologic DMARD that inhibits dihydroorotate dehydrogenase, a key enzyme in pyrimidine synthesis. This suppresses activated T-cell proliferation, leading to reduced inflammation and joint damage [33].

Leflunomide: A study of 108,085 patients with seropositive RA assessed whether DMARDs treatment is associated with incident AF. The cohort included 37,915 seropositive RA patients treated with Leflunomide, including 847 patients with AF and 37,068 patients without AF. Leflunomide use was associated with a significantly increased risk of new-onset AF, exhibiting a 1.21 times higher risk of developing AF than non-users (1.21, 95% CI 1.09, 1.33, *p* < 0.001). The study addressed confounding through a nested case–control design and adjustments for insurance type and comorbidities (e.g., hypertension, diabetes, chronic kidney disease, thyroid disease, HF). However, residual confounding from unmeasured factors, such as lifestyle behaviours and RA disease activity, could not be excluded [34].

An experimental study by Jiang et al. suggested that Leflunomide may improve cardiovascular outcomes by modulating lipid metabolism and endothelial function via the DHODH/AMPK pathway. However, its cardioprotective effects remain unclear due to limited clinical evidence [33].

#### 5.1.3. Antimalarials

HCQ is commonly used for the therapeutic management of RA, as well as malaria prevention, and has been associated with arrhythmias by slowing the rate of action potential firing in the sinoatrial node. This effect is thought to result from the structural similarity between HCQ and class IA anti-arrhythmic quinidine, which blocks sodium and potassium channels. This blockade can prolong the QT interval and increase the risk of torsade de pointes, a potentially life-threatening arrhythmia [35].

Recent papers assessing the association between HCQ and arrhythmias in RA patients report contradictory data. However, most seem to highlight a relatively low risk (Table 1).

A systematic review of MEDLINE and Embase through April 2023 evaluated cardiac conduction abnormalities in patients with systemic autoimmune rheumatic diseases using HCQ or chloroquine (CQ). Five studies included RA patients. Meta-analysis showed a significant increase in mean QTc among RA patients on HCQ/CQ (10.29 ms, 95% CI 2.67–17.91), while the pooled odds of prolonged QTc were not significant (OR 1.49, 95% CI 0.91–2.42). Recent reviews emphasize the overlapping roles of IL-1, IL-6, TNF, and Janus kinase-signal transducer and activator of transcription signalling in RA and cardiovascular disease. Proinflammatory cytokines can directly promote arrhythmias and indirectly via systemic effects such as sympathetic activation, potentially contributing to QTc prolongation in RA. It remains essential for clinicians to maintain heightened awareness of potential cardiovascular adverse effects associated with HCQ and CQ use in patients with systemic autoimmune rheumatic disorders [36].

**Table 1:** The most recent studies on the association between hydroxychloroquine use and arrhythmias in patients with rheumatoid arthritis.

Authors/Year	Type of Study	Number of Patients	Data and Conclusions
Mercedes E Quiñones et al. (2023) [37]	Retrospective, propensity score–matched cohort safety study	8852	The current study includes 8852 Veterans with newly diagnosed RA (4426 treated with HCQ and 4426 treated with other DMARDs), balanced on 87 baseline characteristics. The primary outcome was LQTS over 19 years. Incident LQTS was rare, 0.09% vs. 0.11% at 2 years and 0.38% vs. 0.14% at 5 years (HCQ vs. other DMARDs, HR 2.17 over 10 years). HCQ use was not linked to an increased risk of LQTS during the first two years of treatment. A higher risk was reported after five years, though the absolute risk remained low, with a minimal difference between those taking HCQ and non-users. The risk decreased with longer follow-up, supporting the long-term safety of HCQ in RA patients [37].
M Rashedul Hoque et al. (2023) [38]	Retrospective cohort study	23,036	Based on data from 1996 to 2014, the cohort included 11,518 HCQ initiators and non-initiators. Over a follow-up of eight years, 1610 patients in the HCQ group developed arrhythmias and 1646 in the non-HCQ group, with crude incidence rates of 17.5 and 18.1 per 1000 person-years, respectively. HCQ initiation was not associated with an increased risk of arrhythmia [38].
Chien-Hsien Lo et al. (2021) [39]	Retrospective cohort study	2883	This study analyzed a cohort of patients aged ≥20 years with newly diagnosed RA, enrolled between 2000 and 2012. Patients were divided into three groups: non-HCQ (1606 patients; 48 arrhythmia cases), <400 mg HCQ (1079 patients; 29 cases), and >400 mg HCQ (198 patients; 4 cases). HCQ use was not associated with an increased overall risk of cardiac arrhythmias, including ventricular arrhythmias, with adjusted hazard ratios of 0.94 (*p* = 0.800) for <400 mg and 0.70 (*p* = 0.389) for >400 mg, regardless of dose or treatment duration [39].
Duygu Eryavuz Onmaz et al. (2021) [40]	Observational, analytical study	70	The current study showed that Blood levels of HCQ and its metabolites were positively correlated with the QTc interval, which had a mean value of 390 ms. Measured concentrations were as follows: Desethylchloroquine 69.1 ng/mL, Bidesethylchloroquine 253 ng/mL, and Desethylhydroxychloroquine 310 ng/mL, yielding a total metabolite concentration of 641 ng/mL. Therefore, there was a significant relationship between QTc interval and especially blood desethylchloroquine levels [40].

#### 5.1.4. Sulfasalazine

Sulfasalazine is a synthetic drug comprising 5-aminosalicylic acid and sulphapyridine, connected by azo bonds. While it is commonly used as an anti-inflammatory treatment for inflammatory arthritis and inflammatory bowel disease, sulphapyridine itself may also have therapeutic potential specifically for RA. However, the precise molecular pathways through which sulfasalazine exerts its effects in RA are still not fully understood [41].

There are no studies evaluating the use of sulfasalazine and the prevalence of arrhythmias. A study using an Ang II-induced cardiac remodelling mouse model treated with sulfasalazine evaluated blood pressure, cardiac function, as well as Akt phosphorylation in vivo and in vitro. The results indicate that sulfasalazine, independent of its anti-inflammatory properties, exacerbates cardiac dysfunction, hypertrophy, and fibrosis by aggravating Ang II-induced cardiac remodelling through activation of the Akt signalling pathway. Despite these findings, the exact role of sulfasalazine in cardiac remodelling currently remains unclear [42].

### 5.2. bDMARDs and tsDMARDs

The 21st century brought a paradigm shift in the treatment of RA with the introduction of bDMARDs alongside tsDMARDs, complementing csDMARDs. These advances have made clinical remission achievable for most patients, enabling long-term prevention of joint damage and physical disability progression. The marketed biologics include five TNF-targeting drugs, two IL-6 receptor-targeting drugs, one B cell antigen CD20-targeting antibody, and one selective T cell co-stimulatory modulator [43]. The cardiovascular implications of these agents, along with their correlation with arrhythmic events, are presented in Table 2.

TNF inhibitors are associated with an increased risk of adverse outcomes in patients with pre-existing HF. The 2021 ACR guidelines recommend the use of non-TNF bDMARDs or targeted tsDMARDs in patients with NYHA class III–IV HF or in those with a left ventricular ejection fraction <50%. Among patients with RA and advanced HF who exhibit an inadequate response to csDMARDs, non-TNF bDMARDs or tsDMARDs are preferred over TNF inhibitors [44]. The 2003 Anti-TNF-α Therapy Against Congestive Heart Failure trial randomized 150 patients with advanced HF (NYHA III, EF < 35%) to placebo or infliximab (5 or 10 mg/kg). Although early improvements in inflammatory markers and LVEF were noted at week 14, benefits were not sustained, and by week 28, infliximab was associated with increased adverse outcomes, including hospitalizations and deaths, indicating potential dose-related cardiotoxicity. The 2002 RENEWAL programme enrolling 3171 HF patients (NYHA II–IV, LVEF ≤ 30%) across 25 countries. Patients received placebo or etanercept 25 mg once, twice, or thrice weekly. At 24 weeks, no significant difference in clinical status was observed These recommendations underscore the need for a personalized approach that involves cardiovascular risk assessment, QT interval evaluation, and consideration of concurrent cardiac conditions or cardiovascular disease history to ensure safe and effective RA management [33].

Elevated IL-6 levels in systemic inflammation are linked to acquired LQTS and increased risk of torsade de pointes. While the underlying mechanisms remain unclear, most cases of acquired LQTS involve dysfunction of the hERG channel, which mediates the rapid delayed rectifier K^+^ current (IKr), essential for cardiac repolarization [45]. This suggests that IL-6 inhibitors such as tocilizumab may have a potential antiarrhythmic effect, normalizing the QTc interval by dampening systemic inflammation [46].

CD20 is a transmembrane protein found on B lymphocytes, involved in their activation and differentiation. Rituximab targets CD20 and induces cell death through complement activation, antibody-dependent cytotoxicity, and apoptosis. Following B-cell lysis, CD20 can redistribute to tissues such as cardiac myocytes, where its inhibition may disturb calcium handling and trigger early after depolarisations, potentially leading to arrhythmias like polymorphic VT or torsade de pointes, especially in patients with prolonged QT intervals. However, the exact mechanisms linking rituximab to arrhythmias are still not fully elucidated [47].

The main representative of selective T-cell co-stimulation inhibitors is abatacept. This biologic agent acts by blocking the co-stimulatory CD80/CD86:CD28 pathway, resulting in inhibition of full T cell activation with consequent mitigation of inflammatory processes [48]. Current cardiovascular evidence for abatacept predominantly addresses HF, arterial stiffness, lipid metabolism, and major adverse cardiovascular events, with limited available data on cardiac rhythm disturbances [49]. 

TsDMARDs, such as the JAK inhibitors, represent an advanced class of oral therapies that inhibit JAK enzymes involved in immune signalling. By precisely modulating these pathways, they effectively reduce inflammation and provide alternatives for patients unresponsive or intolerant to conventional or biologic DMARDs. Given their impact on critical signalling pathways, JAK inhibitors require careful monitoring for potential infections and other adverse effects [50]. 

**Table 2:** The cardiovascular effects of tsDMARDs and biologics, along with their reported associations with arrhythmic events (CV, cardiovascular; ELISA, enzyme-linked immunosorbent assay; HF, heart failure; JAK, Janus kinase; LV, left ventricular; MTX, methotrexate; PCR, protein chained reaction; RA, rheumatoid arthritis; TNF, tumour necrosis factor).

**TNF-A inhibitors**	**Etanercept**	**Key Message**	**Supporting Data**
		Cardioprotective in patients without HF. Reduces arterial stiffness, LV mass index, and overall CV morbidity in RA.	Seventy Sprague-Dawley rats with collagen-induced arthritis were assessed (LV structure and function by echocardiography, inflammatory markers by ELISA, and gene expression by quantitative PCR). Etanercept was administered for 6 weeks post-arthritis onset. Systemic inflammation was found to contribute to LV fibrosis and extracellular matrix remodelling by increasing macrophage infiltration and local cardiac expression of pro-fibrotic genes. Etanercept partially inhibited collagen remodelling, but it did not stop diastolic dysfunction from occurring, suggesting that other mechanisms besides TNF-α are involved [51]. Over 6 months of follow-up, etanercept was shown to be safe in terms of cardiac function and lipid profile and to be beneficial in improving RA parameters in patients who had active disease [52].
	Infliximab	May be associated with life-threatening tachyarrhythmia and bradyarrhythmia. Improves arterial stiffness and vascular function in RA patients.	Infliximab and the risk of arrhythmias in RA have not been linked in any recent study. Older data and sporadic case reports provide the only evidence of arrhythmogenicity. Infliximab may be linked to bradyarrhythmia and tachyarrhythmia, which are potentially fatal. The total incidence of arrhythmias during infliximab infusion did not differ significantly from that of a placebo. Ventricular tachyarrhythmias with recent onset, however, were more common and severe. Prolonged QT intervals and decreased heart rate variability were seen in affected patients, primarily those with RA [17].
	Adalimumab	Inconclusive evidence. One study showed increased thrombotic events, but no study confirmed an arrhythmic risk.	A retrospective pharmacovigilance study revealed that adalimumab was the only TNF-α inhibitor associated with an elevated risk of cardiovascular adverse events (myocardial infarction, arterial thrombosis), whereas the other four TNF-α inhibitors did not show any increased risk [53].
	Certolizumab pegol	Isolated case reports of arrhythmias. Favourable CV profile due to reduced systemic inflammation.	A 2016 case report described two RA patients on certolizumab and MTX who developed serious arrhythmias: one with persistent AF resistant to cardioversion, and another with AFl managed by beta-blockers. Certolizumab pegol dosing intervals were extended, and MTX reduced in one case, without RA flare-ups [54].
	Golimumab	Cardioprotective effects in the GO-BEFORE and GO-FORWARD trials through improved cardiovascular markers–however, caution is advised in patients with HF.	A 2014 study evaluating intravenous golimumab (2 mg/kg) plus MTX over 52 weeks in active RA reported one case of AF between weeks 24 and 52 [55]. Across multiple long-term extension studies (GO-AFTER, GO-MORE, GO-FURTHER), no further increase in arrhythmia incidence was observed beyond the isolated previously reported cases [56].
**Interleukine-6 Inhibitors**	Tocilizumab	Potential antiarrhythmic effect—normalizes the QTc interval by dampening systemic inflammation.	QTc normalization was observed following tocilizumab treatment. The mean baseline QTc was longer in RA patients than in controls (422 vs. 412 ms; *p* < 0.001) and decreased to 406 ms post-treatment, becoming significantly lower than in controls. 12 RA patients initially had QTc ≥ 440 ms versus none of the controls (*p* = 0.018), and only one remained above this threshold after treatment. Normalization occurred in both male and female patients, and its correlation with CRP reduction underscores the role of systemic inflammation in cardiac repolarization abnormalities in RA [46].
**CD-20 monoclonal antibody**	Rituximab	May be associated with arrhythmias.	Specific research on the frequency of arrhythmias linked to the use of rituximab for RA is lacking in the literature. Cardiovascular toxicities, such as monomorphic and polymorphic ventricular tachycardia, supraventricular tachycardia, trigeminy, bradycardia, AF, and nonspecific dysrhythmias, have been associated with its widespread use. These effects are thought to be influenced by CD20’s modulation of calcium ion channel function [47].
**Selective T-cell co-stimulation inhibitor**	Abatacept	Most of the currently available data on cardiovascular safety address HF, arterial stiffness, lipid profiles, and major adverse cardiovascular events, rather than cardiac rhythm disorders.	It may slow atherosclerosis progression and benefit high-risk patients [49]. Hypertension has been reported as a possible side effect in 1–10% of cases. However, the cardiovascular risk reduction associated with the anti-inflammatory effect of abatacept appears to remain unaffected [57].
**JAK inhibitors**	Tofacitinib	May increase the risk of cardiovascular adverse events, particularly in older patients or those with pre-existing heart conditions. No current evidence of a direct link between JAK inhibitors and specific arrhythmias.	Tofacitinib demonstrated signals for thromboembolic events and hypertension [58]. The most recent STAR-RA study did not provide evidence of a direct link between tofacitinib and specific arrhythmias. The available data focus solely on major adverse cardiovascular events (including myocardial infarction and stroke) [59].
	Baricitinib		Baricitinib showed stronger associations with thromboembolic events, torsade de pointes/QT prolongation, pulmonary hypertension, ischemic heart disease, cardiac arrhythmias, and cardiac failure [58]. Arrhythmia, HF, and sudden cardiac death have been reported in post-marketing settings [60].
	Upadacitinib		Upadacitinib was associated with pulmonary hypertension, thromboembolic events, ischemic heart disease, torsade de pointes/QT prolongation, HF, cardiac arrhythmias, and cardiomyopathy [58]. It has shown an arrhythmia signal in spontaneous reporting systems, but clinical studies have not confirmed or quantified this risk [61].
	Filgotinib		Integrated analyses of over 12,500 patients with years of exposure confirm low rates of major adverse cardiovascular events and venous thromboembolism, with no evidence of rhythm-related cardiac events [62]. A 2019 study did not find clinically relevant correlations between QTc interval and plasma concentrations of filgotinib or its major metabolite [63].

### 5.3. Complementary Therapies

#### 5.3.1. Corticosteroids

The efficacy of glucocorticosteroids (GCS) has been demonstrated in the therapeutic management of RA. However, challenges posed by dose tapering led to long-term use in up to one-third of patients. Notably, the 2021 ACR guidelines did not recommend the use of GCS as short-term bridge therapy, contrasting with other guidelines that support or offer more nuanced recommendations regarding their use [64]. Steroid therapy may induce a range of arrhythmias through its direct effects on cardiac membrane potassium channels and the regulation of their expression [65].

The literature review conducted by Tisdale et al. analyzed the most commonly used medications that may induce or exacerbate various types of arrhythmias. The incidence of AF and AFl associated with methylprednisolone use was approximately 1.8%. However, the association between corticosteroid use and AF remains inconsistent, as arrhythmias are often secondary to underlying medical conditions rather than the medication itself. Atrioventricular nodal re-entrant tachycardia has also been reported in association with methylprednisolone use, although rare. The mechanism remains unclear but is thought to involve intracellular electrolyte imbalances and repolarisation disturbances [66]. 

The association between corticosteroids and arrhythmogenesis was investigated using male Sprague-Dawley rats. The study found that glucocorticoid administration leads to significant alterations in the expression of key ion channel genes, including those encoding L-type calcium channels and T-type calcium channels, which are crucial for maintaining normal cardiac rhythm. These changes in gene expression result in disrupted calcium ion handling and increased susceptibility to AF. Furthermore, the study highlights the role of the glucocorticoid receptor in mediating these effects, suggesting that glucocorticoid-induced arrhythmogenesis is a receptor-dependent process. The findings underscore the importance of considering glucocorticoid therapy as a potential risk factor for atrial arrhythmias, particularly in patients with underlying cardiac conditions [67]. 

#### 5.3.2. Sinomenine

Sinomenine, the main alkaloid derived from *Sinomenium acutum*, is a traditional Chinese medicine widely used in rheumatology and has shown cardioprotective and anti-arrhythmic effects. It modulates cardiac electrophysiology by prolonging action potential duration, enhancing repolarization, and reducing excitability through inhibition of key ion currents, including L-type calcium, delayed rectifier potassium, IK1, and fast sodium currents. These actions lower intracellular calcium, limit calcium overload, and reduce the risk of arrhythmias, highlighting sinomenine as a potential therapeutic option for cardiac rhythm disorders [68]. 

## 6. Diagnostic of Arrhythmias in Rheumatoid Arthritis

**Figure 4:**
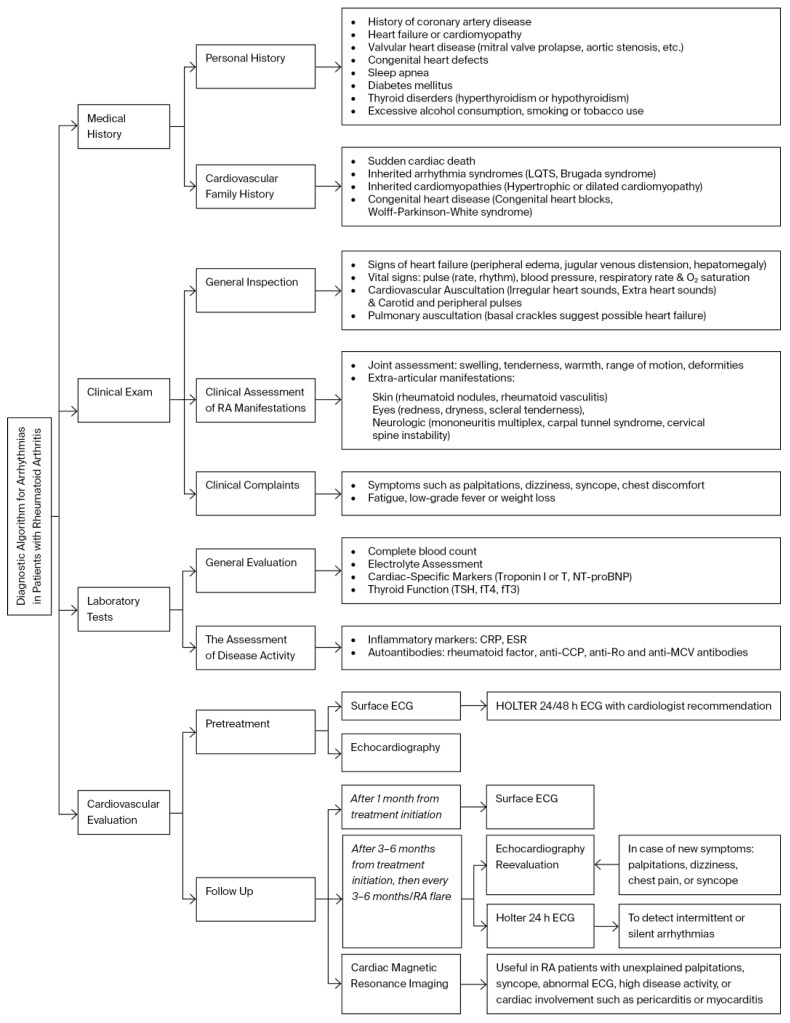
A visual representation of the diagnostic algorithm for arrhythmias in rheumatoid arthritis. (RA, rheumatoid arthritis; CRP, C reactive protein; ECG, electrocardiogram; ESR, erythrocyte sedimentation rate).

Early and accurate diagnosis of cardiac electrophysiology abnormalities in patients with RA is essential, as cardiac involvement may often be subclinical. A multidisciplinary evaluation by rheumatologists and cardiologists is recommended for all RA patients to identify the risk of severe complications, including sudden cardiac death. Initial assessment includes clinical evaluation, laboratory tests, 12-lead ECG, and standard echocardiography, but screening for arrhythmias can be extended [7]. A visual representation of the proposed diagnostic algorithm is presented in Figure 4.

Rheumatoid arthritis has been linked to various subtle abnormalities on the 12-lead ECG. While a normal ECG reflects rapid and synchronous ventricular depolarization and repolarization, patients with RA may exhibit disturbances in both phases of the cardiac action potential. Although some studies report similar QTc durations but increased QT dispersion (QTd), indicating subclinical repolarisation abnormalities, patients frequently present with a corrected QT interval (QTc) prolonged by 10–20 ms in comparison to healthy controls. These discrepancies could be caused by small sample sizes or could be explained by the impact of disease severity and duration. One study observed elevated QTc over a 12-year period in RA patients, but no differences in QTc at diagnosis. Longer QTc durations have been linked to increased CRP levels and cytokine levels, especially IL-10 [9].

Gülkesen et al. identified new ECG indicators that may predict arrhythmias in RA patients. The study compared P-wave dispersion (Pd), corrected QT (QTc) dispersion, Tp-e dispersion, and the Tp-e/QTc ratio between RA patients and healthy controls. P-wave dispersion (Pd) represents the difference between the longest (P max) and shortest (P min) P-wave duration on the ECG. Tp-e defines the interval from the T-wave peak to its end. In RA, local atrial complement activation leads to tissue injury, myocyte necrosis, and atrial fibrosis, resulting in structural remodelling. This creates electrical inhomogeneity, slower conduction, and variable refractory periods, which manifest as prolonged P waves and increased Pd, a known risk factor for paroxysmal and persistent AF. Corrected QT dispersion (QTc d) is defined as the difference between the maximum (QTc max) and minimum (QTc min) QT intervals. Increased QTc d reflects heterogeneous ventricular repolarization, which predisposes to ventricular instability, re-entry, and sudden cardiac death. The Tp-e interval, measured from the peak to the end of the T wave, reflects transmural dispersion of ventricular repolarization. Elevated Tp-e dispersion and Tp-e/QTc ratio indicate a higher risk for ventricular arrhythmias. The study concluded that RA patients showed significantly higher Pd, QTcd, Tp-ed, and Tp-e/QTc values compared to controls, suggesting an increased risk for both atrial and ventricular arrhythmias [1].

Twenty-four-hour Holter ECG offers a more detailed and prognostically meaningful assessment of QTc duration and arrhythmias. This renders it a better option to complete the cardiovascular assessment of RA patients compared to short-term ECG recordings. A study that analyzed 58 RA patients used both short ECG and 24 h Holter monitoring, revealing significantly higher QTc values. The number of QRS complexes with QTc > 450 ms was strongly associated with ventricular ectopy and inflammation, proving that Holter monitoring provides a more accurate evaluation of QTc dynamics and arrhythmic risk in RA patients [69]. 

Both classic and advanced diagnostic tools are valuable for evaluating cardiac function and detecting myocardial involvement and coronary artery disease, key factors in arrhythmias in autoimmune rheumatic diseases. Advanced echocardiographic techniques like 2D/3D speckle-tracking and global longitudinal strain can detect early ventricular dysfunction, diastolic abnormalities, and valvular disease. However, a normal echocardiographic examination does not exclude cardiac involvement. PET and cardiac magnetic resonance offer superior tissue characterization for detecting inflammation and fibrosis [7].

Cardiac magnetic resonance has revealed proarrhythmic substrates in RA, with 45% of asymptomatic patients showing abnormalities, mainly epicardial late gadolinium enhancement, indicative of myocardial fibrosis or myocarditis. Some also showed perfusion defects suggesting microvascular ischemia, possibly due to underlying vasculitis. Late gadolinium enhancement presence correlated with disease activity markers and brain-derived natriuretic peptide levels, indicating subclinical myocardial involvement. These findings suggest that many RA patients may have undetected myocardial fibrosis, and some may benefit from intensive disease-modifying therapy to improve perfusion [9].

## 7. Management of Arrhythmias in Rheumatoid Arthritis

Current strategies for managing cardiac arrhythmias entail a multidisciplinary approach, aiming to optimize outcomes by balancing potential benefits against associated risks of available therapeutic interventions. The treatment plan involves appropriate control of disease activity by csDMARDs/bDMARDs, anti-arrhythmic drugs, autonomic modulation, use of implantable cardioverter–defibrillators (ICD), cardiac stereotactic body radiotherapy (SBRT), and catheter ablation [70]. 

**Figure 5:**
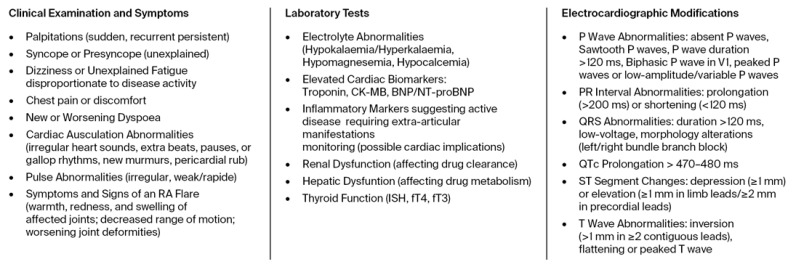
Illustration of arrhythmia risk red flags in rheumatoid arthritis patients (RA, rheumatoid arthritis).

The use of csDMARDs and bDMARDs has raised concerns regarding their cardiovascular safety, including the risk of cardiac arrhythmias. While traditional DMARDs (e.g., MTX, sulfasalazine) generally have a favourable safety profile, emerging data suggest that some bDMARDs may influence electrophysiological stability. The clinical, biological, and electrocardiographic indicators of arrhythmia risk in RA patients proposed as red flags are synthesized in Figure 5.

Anti-arrhythmic drugs are pharmacologic agents used to treat abnormal cardiac rhythms by modifying electrical impulses within the myocardium. They are typically classified according to the Vaughan-Williams classification, which categorizes them based on their primary electrophysiological effect. However, considering their potential adverse effect and the autoimmune nature of RA, therapeutic strategies must be approached with heightened clinical vigilance and tailored to the individual patient’s risk profile. The relevant side effects of anti-arrhythmic drugs are summarized in Table 3. Moreover, drug–drug interactions between anti-arrhythmic agents and conventional, biologic, or targeted synthetic DMARDs must be carefully monitored, especially with agents metabolized via the hepatic cytochrome P450 pathways [71]. 

Rheumatoid arthritis patients often encounter limitations with antiarrhythmic drugs for treating AF due to adverse effects, reduced efficacy, and condition-specific contraindications. Catheter ablation with pulmonary vein isolation represents an effective alternative for AF management, regardless of the presence of structural heart disease. While studies indicate that catheter ablation is generally safe and reasonably effective in RA patients, evidence regarding the risk of atrial tachyarrhythmia recurrence after the procedure remains inconsistent. A systematic search of Medline and EMBASE up to December 2023 identified studies comparing AF recurrence after catheter ablation in patients with and without RA. Three retrospective cohort studies (total *n* = 700) were included. Meta-analysis using a random-effects model showed RA patients had a higher risk of AF recurrence after catheter ablation (pooled RR 1.59, 95% CI 1.10–2.29) and early recurrence within 90 days (pooled RR 2.70, 95% CI 1.74–4.19). These results suggest that RA is linked to a higher risk of both overall and early AF recurrence following catheter ablation. The chronic inflammatory state in RA may drive extensive atrial remodelling and fibrosis, generating a more complex atrial substrate that predisposes patients to recurrence [72]. 

**Table 3:** Side effects of anti-arrhythmic drugs relevant in RA patients (AF, atrial fibrillation; APD, action potential duration; AV, atrio-ventricular; JAK, Janus kinase; MI, myocardial infarction; PSVT, paroxysmal supraventricular tachycardia; SVT, supraventricular tachycardia; VF, ventricular fibrillation; VT, ventricular tachycardia; WPW syndrome, Wolf-Parkinson-White Syndrome).

Class	Mechanism of Action and Indications	Examples	Safety Considerations in RA
**Class I (Sodium channels blockers)**			
**Ia**	Slows conduction, prolongs repolarization. Used for AF, VT, WPW syndrome.	Quinidine, Procainamide, Disopyramide	Due to the potential of quinidine to cause lupus-like syndrome, its use should be carefully evaluated [73]. Irizarry-Caro reported that procainamide is a lupus-inducing drug, promoting NET formation via neutrophil muscarinic receptor activation and calcium flux, highlighting innate immune involvement in DILE [74]. Rhupus syndrome, an overlap of RA and systemic lupus erythematosus, occurs in approximately 0.01–2% of rheumatic disease cases and the use of lupus-inducing drugs needs to be restricted [75]. There are no reports about autoimmunity induced by disopyramide or side effects that can impact RA patients.
**Ib**	Shortens repolarization. Used for VT (especially post-MI), not for atrial arrhythmias.	Lidocaine, Mexiletine	Lidocaine is metabolized in the liver. This is especially relevant, as RA patients often use hepatotoxic drugs such as MTX, which can impair liver function and reduce lidocaine clearance, increasing the risk of toxicity [76]. Mexiletine use is associated with various side effects, mostly related to the gastrointestinal and nervous system, but also hematological. The most prevalent symptoms are nausea, abdominal pain or discomfort, tremors, headaches, dizziness, and thrombocytopenia [77].
**Ic**	Slows conduction, no effect on repolarization. Used for AF, SVT.	Flecainide, Propafenone	Patients with RA are at elevated risk for interstitial lung disease. Flecainide has been linked to drug-induced interstitial pneumonitis or diffuse alveolar damage [78]. Propafenone is recognized as a very low-risk drug for drug-induced lupus, with <0.1% incidence for routinely used doses [73].
**Class II (β-adrenergic receptor blockers)**			
**Beta-blockers reduce sympathetic tone and slow atrioventricular (AV) conduction.Used for AF (rate control), SVT, ventricular ectopy, post-MI.**		Metoprolol, Atenolol, Esmolol	Beta-blocker use was independently associated with a lower likelihood of achieving remission [79]. In 1986, FDA reports described several cases of joint pain linked to metoprolol, which resolved within a few days after drug administration was ceased [80]. Atenolol can cause vasculitis and drug-induced lupus erythematosus [81]. There are no studies on the effects of Esmolol on RA disease activity.
**Class III (Potassium channel blockers)**			
**Prolongs repolarization (increasing APD and QT interval).** **Used for AF, VT, VF.**		Amiodarone, Sotalol, Dofetilide, Ibutilide	Patients with preexisting pulmonary disease are particularly vulnerable to amiodarone, with reports of diffuse alveolar hemorrhage syndrome [82]. Sotalol can induce vasculitis [81]. Dofetilide is primarily excreted through the kidneys, while approximately 20- 30% is metabolized in the liver via the CYP3A4 enzyme pathway. Therefore, dose adjustments are necessary for RA patients with impaired kidney function [83]. There are no reports of Ibutilide on RA disease activity.
**Class IV (Calcium channel blockers)**			
**Decreases AV node conduction and automaticity.** **Used for AF (rate control), SVT.**		Verapamil, Diltiazem	Calcium channel blockers, specifically diltiazem and verapamil, have been well established as strong inhibitors of CYP3A4, with most major drug interactions linked to these two medications [84]. Tofacitinib, a JAK inhibitor, is also metabolized by the CYP3A4 pathway. In these cases, co-administration needs to be carefully evaluated [85].
**Others**			
**Mixed or unique mechanisms**. **Used for PSVT (adenosine), rate control in AF (digoxin), sinus tachycardia.**		Adenosine, Digoxin, Ivabradine	Digoxin was well tolerated in RA patients and demonstrated significant immunomodulatory and anti-inflammatory effects. Additionally, it may possess anti-angiogenic properties, suggesting that it could serve as an effective complementary therapy to csDMARDs in the treatment of RA [86]. There are no studies about Adenosine or Ivabradine linked to negative effects on RA disease activity.

Alternative approaches are needed to alter arrhythmic substrates without further damaging heart tissue. Reducing procedure-related inflammation through targeted therapies may lower arrhythmia recurrence. Cell-based molecular therapies show promise in restoring gene expression, protecting heart cells, regulating immune responses, and minimizing scarring to improve heart function [87]. 

Autonomic nerve remodelling, involving vagal stimulation that shortens the atrial refractory period, is a potential gene therapy target. Inhibiting components of the G-protein coupled autonomic pathway (like Gαi and Gαo) has effectively modulated atrial electrophysiology and reduced AF in animal studies. Gap junction remodelling, due to decreased or mis-localized connexins (Cx40, Cx43), disrupts electrical conduction and raises arrhythmia risk. Targeting these connexins improves conduction and lowers arrhythmia burden. Structural remodelling, characterized by fibrosis and increased TGF-β, is another key target; blocking TGF-β signalling can reduce fibrosis and subsequent arrhythmias. For catecholaminergic polymorphic ventricular tachycardia, gene-based strategies such as gene replacement, mutant gene silencing, CRISPR/Cas9 editing, and pathway suppression have shown promise in preclinical models [70]. 

## 8. Future Perspectives of Arrhythmias in Rheumatoid Arthritis

Future perspectives for the management of arrhythmias in RA increasingly focus on gene-based therapies. These approaches aim to identify and mitigate the genetic mechanisms underlying AF [87]. Two primary mechanisms driving AF are focal ectopic firing and re-entry, both influenced by electrical and structural remodelling, autonomic nervous system alterations, and abnormalities in calcium handling [22].

Electrical remodelling is typically characterized by a shortening of the atrial action potential duration, which results from a reduction in the L-type calcium current and an increase in the inward-rectifier potassium current. Furthermore, constitutive activation of the acetylcholine-induced potassium current may also contribute to this process. Abnormal calcium handling is another key contributor to ectopic activity, particularly due to calcium leak from the sarcoplasmic reticulum, via the ryanodine receptor type 2. Targeting specific phosphorylation sites on ryanodine receptor type 2 or employing modified forms of calmodulin has demonstrated potential in attenuating sarcoplasmic reticulum calcium leak and reducing the susceptibility to AF [87]. 

Considering the restricted availability of publications addressing this topic, current knowledge is constrained by heterogeneous methodologies, variable findings, and significant gaps in evidence. Future research should therefore adopt more rigorous and standardized designs to clarify the associations between RA and arrhythmia risk. The development of dedicated cardio-rheumatology teams may provide a valuable framework for fostering multidisciplinary collaboration and generating more clinically relevant data.

## 9. Conclusions

Rheumatoid arthritis is recognized not only as a joint-destructive autoimmune disease but also as a systemic inflammatory rheumatic disorder with significant cardiovascular implications. Epidemiological data showed an elevated incidence of cardiac arrhythmias, particularly AF and conduction abnormalities due to chronic inflammation, structural cardiac remodelling, and autonomic dysfunction. The presence of comorbidities such as interstitial lung disease, vasculitis, and ischemic heart disease further heightens this risk.

Early identification of arrhythmias in RA is often complicated by overlapping symptoms (e.g., fatigue, dyspnea) and subclinical manifestations. Proactive cardiovascular screening, which includes echocardiography, Holter analysis, and ECG monitoring, is therefore essential, particularly in patients with a history of heart disease or cardiac involvement and highly active rheumatic disease. A multidisciplinary approach is required for the therapeutic management of arrhythmias in RA, adjusting treatment to each patient’s risk profile while accounting for drug–drug interactions with biologics or DMARDs.

The previously published reviews refer to a specific arrhythmia (often AF) and do not detail the clinical issues associated with their diagnosis and therapy. We believe that our manuscript, prepared by a team of experienced cardiologists and rheumatologists, includes essential data for the rheumatologist and cardiologist clinician in clinical practice and emphasizes the need for this team to follow these patients.

Optimal arrhythmia management in RA hinges not only on cardiologic intervention but also on effective control of systemic inflammation. Emerging evidence suggests that certain bDMARDs may indirectly reduce arrhythmic burden by attenuating inflammatory pathways implicated in atrial and ventricular remodelling. Further research is warranted to define the precise interplay between immunomodulation and cardiac electrophysiology in this population.

## Data Availability

No new data were created or analyzed in this study.

## Abbreviations

The following abbreviations are used in this manuscript:

β1AR	Β1-Adrenergic Receptor
ACE	Angiotensin-Converting Enzyme
ACE 2	Angiotensin-Converting Enzyme 2
ACPA	Anti-Citrullinated Protein Antibodies
AF	Atrial Fibrillation
AFl	Atrial Flutter
Ang II	Angiotensin II
anti-MCV	Anti-Modified Citrullinated Vimentin Antibodies
bDMARDs	Biologic Disease-Modifying Antirheumatic Drugs
cGMP	Cyclic Guanosine Monophosphate
csDMARDs	Conventional Synthetic Disease-Modifying Antirheumatic Drugs
CV	Cardiovascular
CQ	Chloroquine
DILE	Drug-Induced Lupus Erythematosus
EAT	Epicardial Adipose Tissue
ELISA	Enzyme-Linked Immunosorbent Assay
E-selectin	Endothelial Selectin
ESR	Erythrocyte Sedimentation Rate.
GCs	Glucocorticoids
GRK2	G Protein-Coupled Receptor Kinase 2
HCQ	Hydroxychloroquine
HF	Heart Failure
ICAM-1	Intercellular Adhesion Molecule-1
ICD	Implantable Cardioverter–Defibrillator
IKr	Rectifier K^+^ Current
JAK	Janus Kinase
LA	Left Atrial
LEF	Leflunomide
LQTS	Long QT Syndrome
LV	Left Ventricular
MCP-1	Monocyte Chemoattractant Protein-1
MHCII	Major Histocompatibility Complex Class Ii
MTX	Methotrexate
RyR2	Ryanodine Receptor Type 2
NETs	Neutrophil Extracellular Traps
NSAIDs	Nonsteroidal Anti-Inflammatory Drugs
Pd	P-Wave Dispersion
PKG	Protein Kinase G
PSVT/SVT	Paroxysmal Supraventricular Tachycardia/Supraventricular Tachycardia
QTcd	Corrected Qt Dispersion
QTd	QT Dispersion
RA	Rheumatoid Arthritis
RAAS	Renin–Angiotensin–Aldosterone System
RAS	The Renin–Angiotensin System
RF	Rheumatoid Factor
SBRT	Cardiac Stereotactic Body Radiotherapy
tsDMARDs	Targeted Synthetic Disease-Modifying Antirheumatic Drugs
VCAM-1	Vascular Cell Adhesion Molecule-1
VT	Ventricular Tachycardia
WPW	Wolff-Parkinson-White Syndrome
